# Missed and true interval cancers on digital breast tomosynthesis screening mammograms: radiologists’ assessment and artificial intelligence markings

**DOI:** 10.1177/20584601261467016

**Published:** 2026-08-07

**Authors:** Nataliia Moshina, Marthe Larsen, Hildegunn S Aase, Kerstin Obst-Gleditsch, Ingrid Margrethe Ohm Ulvik, Hauke Bartsch, Leif Oltedal, Solveig Hofvind

**Affiliations:** 1 Department of Breast Cancer Screening, Cancer Registry, 25563 Norwegian Institute of Public Health, Oslo, Norway; 2 Department of Radiology, 60498 Haukeland University Hospital, Bergen, Norway; 3 Mohn Medical Imaging and Visualization Centre (MMIV), Department of Radiology, 60498 Haukeland University Hospital, Bergen, Norway; 4 Department of Clinical Medicine, University of Bergen, Bergen, Norway; 5 Department of Health and Care Sciences, Faculty of Health Sciences, UiT, The Arctic University of Norway, Tromsø, Norway

**Keywords:** digital breast tomosynthesis, radiological review, screening, interval cancer, artificial intelligence

## Abstract

**Background:**

Interval cancer, breast cancer detected after a negative screening examination but before the next scheduled appointment, represents a challenge in mammography screening programs due to less favorable histopathological characteristics compared to screen-detected cancer.

**Purpose:**

To determine which interval cancers from digital breast tomosynthesis (DBT) were classified as missed and true by radiologists in a review, and to stratify the findings by risk scores and markings provided by an artificial intelligence (AI) model.

**Material and methods:**

In this retrospective informed consensus-based review, radiologists assessed mammograms from 46 interval cancers and classified those as false negative, minimal-sign significant or non-specific, or true negative. An AI risk score (1–7, low; 8–9, intermediate; or 10, high risk of malignancy) was available for each examination. For cases with AI risk scores of 8–10, the location of AI-detected markings was compared with the true cancer site.

**Results:**

A total of 17% (8/46) of interval cancers were classified as false negative, 22% (10/46) as minimal-sign significant, 20% (9/46) as minimal-sign non-specific, and 41% (19/46) as true negative. The AI model correctly identified 35% (16/46) and incorrectly located 24% (11/46). Considering false negative and minimal-sign significant as cases with the highest probability of being diagnosed at screening due to mammographic visibility, the proportion of cases correctly identified by the AI model was reduced from 35% to 22% (10/46).

**Conclusion:**

About 20% of interval cancers have potential to be diagnosed earlier using AI in DBT screen-reading.

## Introduction

Digital breast tomosynthesis (DBT) has been developed to address the limitations of standard digital mammography (DM) in mammographic screening and breast cancer detection, including overlapping tissue and sufficient contrast for high-density breasts.^1,2^ Meta-analyses comparing the screening performance of DBT and DM have demonstrated a higher screen-detected cancer detection rate for DBT,^3^ as well as lower recall and interval cancer rates for DBT, suggesting overall superior outcomes in cancer detection.^4,5^ However, the reduction in interval cancer rates for DBT relative to DM remains controversial, as some studies have reported no statistically significant differences between the two modalities,^6,7^ while others have observed higher interval cancer rates for DBT.^8^

True-negative interval cancers are shown to have less favorable prognostic and predictive histopathological characteristics compared to false negative interval cancers.^9,10^ Therefore, reducing the number of interval cancers by earlier detection is crucial for reduction of treatment related harms.^11–14^ Retrospective radiological reviews of interval cancers offer a deeper analysis of the mammographic characteristics of these cases, providing valuable learning opportunities that support radiologists in their screen-reading performance.^15^ A systematic review within population-based breast screening programs using screen-film or DM has indicated that 4–40% of interval breast cancer cases were missed by radiologists due to a reader error.^15^ In contrast, radiological reviews following DBT screening are sparse^16–18^ with 5–33% of interval cancers potentially interpreted as false negative, or missed due to a reader error.^16,17^

The use of artificial intelligence (AI) in DM screening has shown promising results for breast cancer detection and the potential to reduce interval cancer rates.^19–22^ Retrospective studies investigating AI-assisted DBT screening have reported acceptable performance metrics compared to standard DM.^2,23^ However, evidence regarding the ability of AI models to correctly identify cancers classified as negative by radiologists during DBT screening remains limited.^17,20^ Furthermore, in AI-assisted screening, the main decision regarding recall is made by radiologists, and mammographic visibility coupled with high suspicion of malignancy of the findings marked with high AI scores could be associated with the highest probability of recall by radiologists.^17,20,22^ Therefore, it is important to understand which interval cancers can be identified by AI but are mammographically invisible for radiologists.

To address these challenges, we analyzed the DBT screening mammograms of interval cancer cases using an AI model and performed an informed consensus-based radiological review of the screening and diagnostic mammograms. Our aim was to determine which interval cancer cases were classified as missed or true by radiologists and to stratify the findings according to AI risk scores and the location of the AI-detected markings.

## Material and methods

In Norway, DBT was used in the Tomosynthesis trials in Bergen (To-Be) as part of the organized mammographic screening program, which invites women aged 50–69 biennially.^24^ In To-Be 1, women were randomly assigned to screening with either DBT or DM between 2016 and 2017, while To-Be 2 included women screened solely with DBT from 2018 to 2019.^25,26^ The interval cancer rate was 0.15% in To-Be 2,^27^ consistent with previous studies using DBT.^28–30^

Screening in To-Be 2 was conducted at the Breast Screening Unit at Danmarksplass in Bergen, as a part of BreastScreen Norway, while screen-reading, recall assessment, and diagnosis were performed at the Breast Diagnostic Center, Haukeland University Hospital, Bergen. Detailed information on the detection and characteristics of screen-detected and interval breast cancers in To-Be 2 has been reported.^24^

All screening examinations were independently read by two radiologists using a 5-point interpretation scale for each breast: 1 indicated a negative result, 2 probably benign, 3 intermediate suspicion, 4 probably malignant, and 5 highly suspicious for malignancy.^31^ Examinations scored 2 or higher by either or both radiologists were discussed in consensus meetings to determine whether the woman should be recalled. All examinations were performed using the Senographe Pristina system (GE Healthcare).

The Regional Committee for Medical and Health Research Ethics approved the study (no. 2018/2574) with a legal basis in Articles 6 (1) (e) and 9 (2) (j) of the General Data Protection Regulation. As the reviewers were internal, no confidentiality statements were needed. All women enrolled in the Tomosynthesis trials provided informed consent. The trials were approved by the Regional Committee for Medical and Health Research Ethics (no. 2015/424).

### Retrospective informed review by radiologists

This internal consensus-based informed review of DBT screening mammograms from interval cancers cases was conducted in August 2025 by three radiologists experienced in mammography reading (HSA; 28 years in screening, whereas 10 with DBT, KOG; 9 and 7 years and IMOU; 17 and 9 years, respectively), with two representatives from the Cancer Registry at the Norwegian Institute of Public Health, recording the results. Interval cancer was defined as breast cancer diagnosed within 24 months after a negatively interpreted screening or 6–24 months following a false positive screening result.^31^

The interval cancers were classified as true negative, minimal-sign non-specific, minimal-sign significant, or false negative by the radiologists. Screening and diagnostic images, biopsy confirmed breast cancer location, as well as histopathology, treatment, and follow-up reports, were available for all cases. True negatives were defined as interval cancer cases with no mammographic findings at the cancer site warranting a recall. Minimal-sign non-specific were defined as cases with non-specific findings at the cancer site, where recall was not indicated. Minimal-sign significant were defined as cases with subtle findings at the cancer site that would not necessarily warrant recall. False negatives were defined as cases with obvious findings at the cancer site, representing potential reader errors.^10,32^

The radiologists reported mammographic density according to BI-RADS 5th edition (*a–d*) and described suspicious lesions at the cancer site using a modified BI-RADS lexicon to classify mammographic features; mass, calcification, asymmetry, and architectural distortion.^33^ Masses were further characterized by shape (oval, round, irregular), margin (circumscribed, obscured, microlobulated, indistinct, spiculated), and density (high, equal, low, fat-containing). Mammographic features were recorded at the review of screening and diagnostic mammograms of cases classified as false negative or minimal-sign significant. The detailed characteristics of masses were provided to enhance radiologists’ knowledge in interpreting DBT mammograms,^20,34^ as accurate characterization of mammographic features is essential in clinical practice.^35^

### AI analysis

The Transpara™ AI model (Version 2.1.0-A, ScreenPoint Medical BV, Netherlands) was applied to all 46 DBT screening examinations from women diagnosed with interval cancers.^36^ The model provided a continuous “AI region score” ranging from 0 to 100 where low risk corresponded to an AI region score of ≤42, intermediate risk to 43–74 and elevated risk ≥75. Further, each examination was assigned a continuous AI raw score from 0 to 10, used to create a categorical AI risk score ranging from 0 to 10,^11,34^ with scores 1–7 considered low risk, 8–9 intermediate risk, and 10 high risk of malignancy. In practical terms, low risk corresponded to AI categorical scores of 1–7 with an AI region score of ≤42, intermediate risk to AI scores of 8, 9 and 10 with an AI region score of <75, and elevated risk to an AI score of 10 with an AI region score of ≥75. Following the radiological review and classification, the locations of AI markings were compared with the true, biopsy confirmed, cancer sites. AI markings were only available for examinations with intermediate or high-risk scores, including AI categorical scores of 8–10, corresponding to AI region scores of >42, consistent with vendor default settings to limit false positives. The AI model was not used for mammographic density assessment.

For interval cancers not visible for the radiologists on DBT screening mammograms, tumor locations were identified using additional modalities, including DM, ultrasound, and magnetic resonance imaging. An AI marking was visible on a mammogram as an AI region score and was considered a “match” if the region with the highest AI region score overlapped with the biopsy confirmed cancer location in craniocaudal (CC) and/or mediolateral oblique (MLO) views. Matching was determined visually by comparing diagnostic images with AI-marked screening mammograms.

### Study sample and histopathological characteristics of cancers

**Figure 1.**
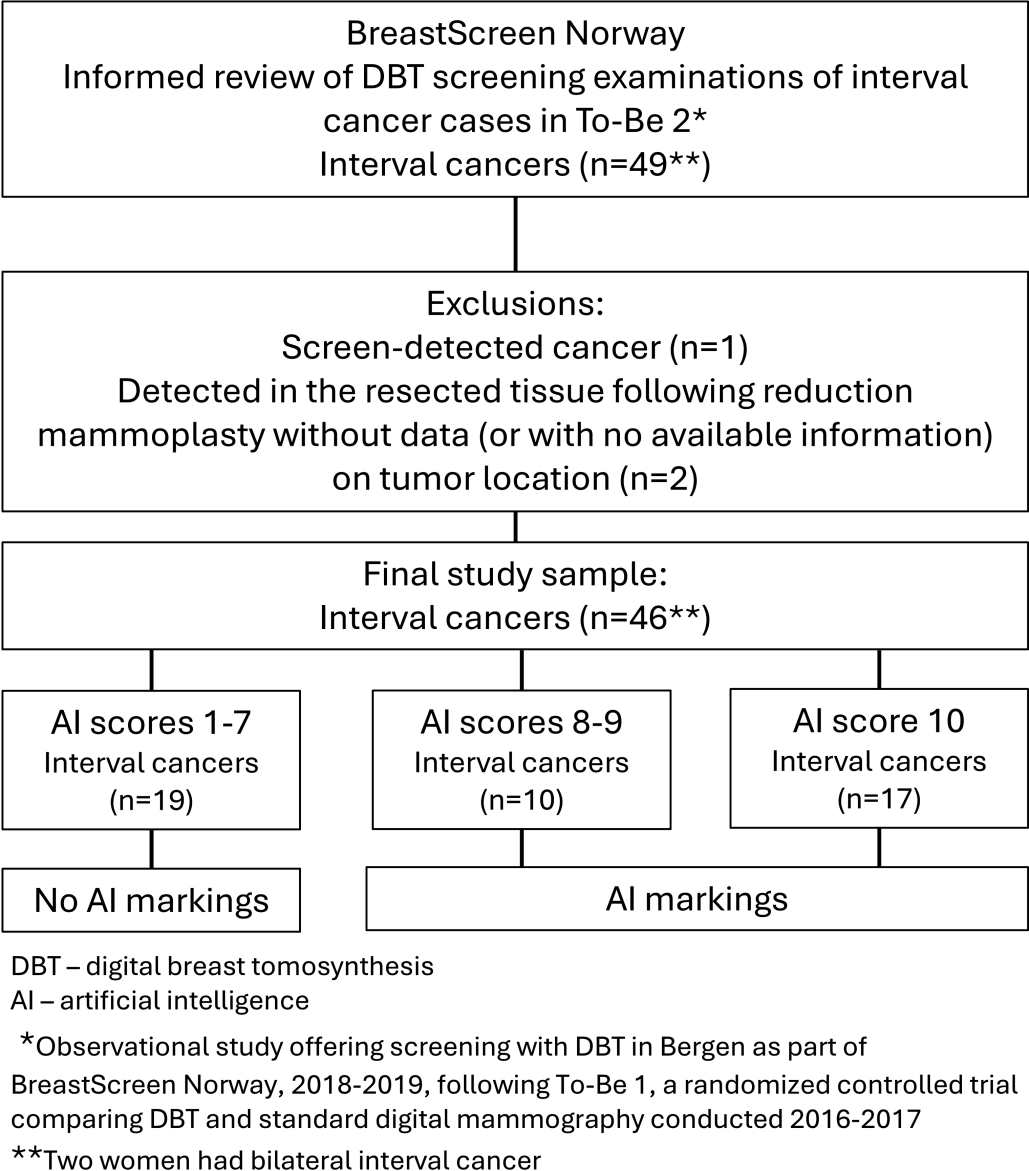
Flowchart of the study sample.

A total of 31,082 women were screened with DBT in To-Be 2,^24^ while 30,724 examinations were run through the AI model. This study obtained information on 49 interval cancers, including ductal carcinoma in situ and invasive breast cancer, that were initially identified in 47 women (Fig. 1). One case of screen-detected cancer misclassified as interval cancer and two cases detected in resected tissue following reduction mammoplasty, with no available tumor location, were excluded. The final study sample for the review consisted of 46 interval cancers.

Data on tumor diameter (mm), histological grade (1-3),^37^ lymph node status (positive or negative), estrogen receptor (ER, positive for >1% expression), and progesterone receptor (PR, positive for ≥10% expression) status, and immunohistochemical subtypes were presented for invasive interval cancers.^38^ Immunohistochemical subtypes were defined based on ER and PR status, human epidermal growth factor receptor 2 (HER2)^39^ expression, and Ki-67, as follows: luminal A (ER+, PR + or PR-, HER2-, Ki-67 ≤ 30 or grade <3 if Ki-67 was absent); luminal B HER2- (ER+, PR + or PR-, HER2-, Ki-67 > 30 or grade 3 if Ki67 was absent); luminal B HER2+ (ER+, PR + or PR-, and HER2+); HER2+ (ER-, PR-, and HER2+); and triple negative (ER-, PR-, and HER2-).^40^

### Statistical analysis

The distribution of interval cancers classified by radiologists as true negative, minimal-sign significant, minimal-sign non-specific, or false negative in the informed consensus-based review was reported alongside AI risk scores (scores 1-7, 8-9, and 10). The radiologists’ classifications were further presented in relation to AI markings with regard to the true cancer location (“match”/“no match”) by mammographic view. Mammographic features and characteristics of the masses were stratified by interval cancer classification and presented in supplementary tables due to the small number of cases in each subgroup. Decimal points were omitted, and no statistical tests were performed because of the limited sample size. Histopathological tumor characteristics were shown. All interval cancers assessed in the review and interval cancers with the correct location of AI markings were presented by months since breast cancer screening (≤12 months vs 13–24 months). Stata (StataCorp. 2023. *Stata Statistical Software: Release 19*. College Station, TX: StataCorp LLC) was used for descriptive tabulations.

## Results

This informed, consensus-based radiological review included 46 interval cancer cases from 44 women, that is, two bilateral interval cancers (Fig. 1). The mean age at diagnosis was 61 years (standard deviation, SD = 5). Mammographic breast density *a* was assigned in 5% (2/44) of the women, *b* in 68% (30/44), *c* in 25% (11/44), and *d* in 2% (1/44). Radiologists classified 41% (19/46) of interval cancer cases as true negative, 20% (9/46) as minimal-sign non-specific, 22% (10/46) as minimal-sign significant, and 17% (8/46) as false negative (Table 1).

**Table 1.** Classification of the interval cancers and malignancy risk score by the artificial intelligence (AI) model (AI score) where 1–7 are considered low risk, 8–9 intermediate risk, and 10 high risk of breast cancer.

​	All cases *n* (%)	AI risk score 1–7 *n* (%)	AI risk score 8–9 *n* (%)	AI risk score 10 *n* (%)	Correct location of the AI marking in CC or MLO for AI risk score 8–10, *n* (%)
True negative^a^	19 (41%)	12 (63%)	3 (30%)	4 (24%)	1 (6%)
Minimal-sign non-specific	9 (20%)	3 (16%)	5 (50%)	1 (6%)	5 (31%)
Minimal-sign significant	10 (22%)	3 (16%)	2 (20%)	5 (29%)	5 (31%)
False negative	8 (17%)	1 (5%)	0	7 (41%)	5 (31%)
Total	46	19	10	17	16

CC—craniocaudal.

MLO—mediolateral oblique.

Total number of cases for each column was denominator for calculating percentages.

^a^Two interval cancer cases that were mammographically occult at diagnosis are included in this group.

Overall, 41% (19/46) of the interval cancers had AI risk scores of 1–7, 22% (10/46) had scores of 8–9, and 37% (17/46) had a score of 10 (Table 1). A total of 43% (13/30) of examinations with BI-RADS density *b* had an AI risk score of 10, while it was 27% (3/11) for density *c*. Among cases with AI scores of 1–7, 63% (12/19) were classified as true negative in the review, whereas 24% (4/17) were classified as true negative among those with an AI score of 10. Conversely, 5% (1/19) of those with AI scores of 1–7 were classified as false negative in the review, compared to 41% (7/17) among those with an AI score of 10. A total of 35% (16/46) were correctly located by AI on screening mammograms, including correct marking in CC or MLO for examinations with AI scores of 8–10 (Table 1).

AI markings were available for 27 interval cancers with an intermediate or high AI score (scores 8–10). The AI model correctly identified the cancer location (highest AI score on a screening mammogram) for 59% (16/27) of these cases (Table 2). Among cases correctly identified by AI, three matched only in the CC view and seven only in the MLO view. For interval cancers with an AI score of 10, 65% (11/17) had a correct location of the AI marking in either CC and/or MLO.

**Table 2.** Matching between the interval cancer location and the location of AI markings for 27 interval cancer cases with AI scores of 8–10. Percentages were provided using the total of 27 cases as denominator.

​	Match in CC only, *n* (%)	Match in MLO only, *n* (%)	Match in both CC and MLO, *n* (%)	Match in CC and/or MLO, *n* (%)	No match, *n* (%)	Total, *n* (%)
True negative	1	0	0	1	6	7 (26%)
Minimal-sign non-specific	0	4	1	5	1	6 (22%)
Minimal-sign significant	1	2	2	5	2	7 (26%)
False negative	1	1	3	5	2	7 (26%)
Total	3 (11%)	7 (26%)	6 (22%)	16 (59%)	11 (41%)	27 (100%)

CC—craniocaudal.

MLO—mediolateral oblique.

**Figure 2.**
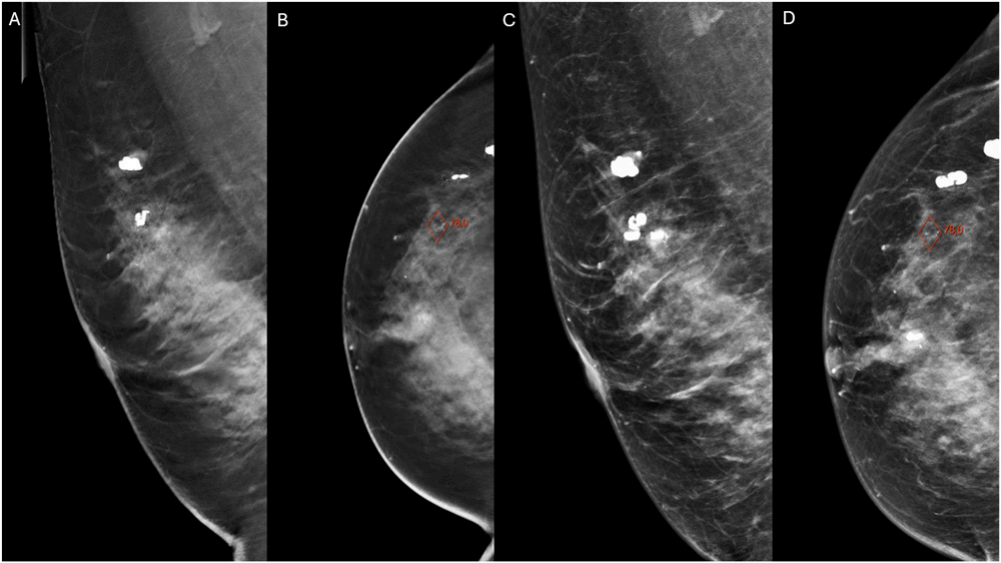
(a) Right mediolateral oblique and (b) craniocaudal digital breast tomosynthesis mammograms at 1-mm plane, and (c) right mediolateral oblique and (d) craniocaudal synthetic two-dimensional mammograms showing an artificial intelligence (AI) marking (red square) in craniocaudal mammograms (b) and (d) corresponding to an AI score of 10, with an AI region score of 78, observed in upper lateral quadrant in a 63-year-old woman at screening in 2019. Interval cancer, 15-mm histologic grade 2 ductal carcinoma in situ, was diagnosed in the AI marked location in 2021. The case was classified as true negative at the retrospective consensus-based informed review.

**Figure 3.**
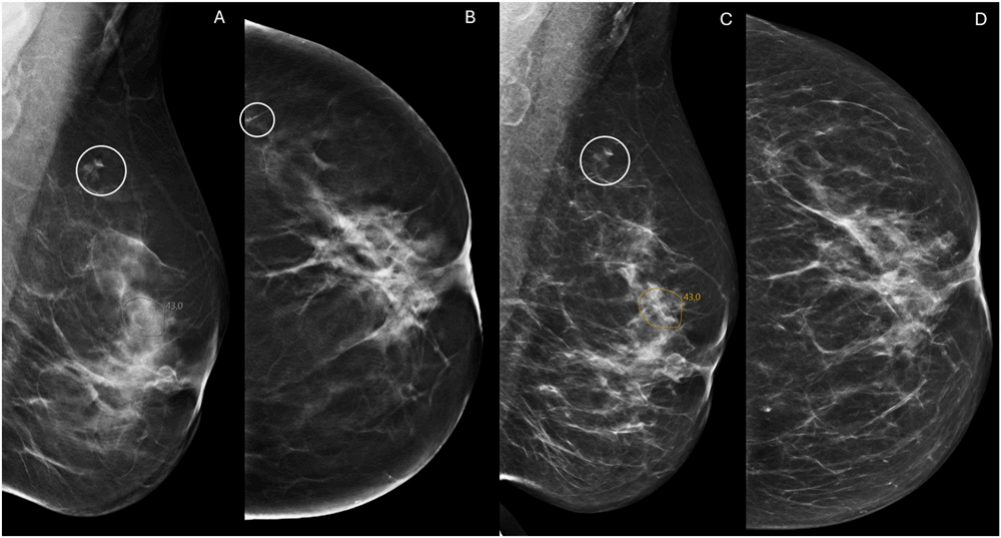
(a) Left mediolateral oblique and (b) craniocaudal digital breast tomosynthesis mammograms at 1-mm plane, and (c) left mediolateral oblique and (d) craniocaudal synthetic two-dimensional mammograms showing a mass (marked with a white circle) in upper lateral quadrant in a 57-year-old woman at screening in 2018. Artificial intelligence (AI) markings in mediolateral mammograms (A, marked with a grey circle, and C, marked with a yellow circle) corresponding to an AI score of 8, with an AI region score of 43, did not match the true cancer location. Interval cancer, 18 mm histologic grade 3 luminal A invasive breast cancer of no special type, was diagnosed in 2019. The case was classified as false negative at the retrospective consensus-based informed review.

Focusing on the radiological review classification, screening mammograms of the true negative case correctly marked by AI (Table 2) are shown in Fig. 2. Five of seven false negative cases with available AI markings had correct AI locations in any of the views (Table 1 and Table 2), corresponding to 11% (5/46) of all interval cancers. Two of seven false negative cases did not have correct AI markings. One of those cases is shown in Fig. 3. Among the minimal-sign significant cases, five of seven had AI markings that matched with the cancer location in CC and/or MLO view. This corresponded to 11% (5/46) of all interval cancers. Combining false negative and minimal-sign significant cases, 22% (10/46) had an intermediate or high AI score and an AI marking corresponding to the true cancer location.

When reviewing screening mammograms, radiologists classified seven of eight false negative and three of ten minimal-sign significant cases as masses (Supplemental Table 1). When reviewing diagnostic images, radiologists classified 10 of 19 true negatives, five of ten minimal-sign non-specific, five of ten minimal-sign significant, and all false negatives as masses. Among the seven false negative cases classified as masses at the review of screening mammograms, five had an irregular shape, five had spiculated margins, and five had equal density (Supplemental Table 2). For the eight false negative cases classified as masses at the review of diagnostic mammograms, seven had an irregular shape, all eight had spiculated margins, and six had high density.

When investigating histopathological tumor characteristics, all invasive interval cancers classified as true negative by the review were histologic grade 2 (72%, 11/15) or 3 (28%, 4/15) cancers (Supplemental Table 3). For cases with available AI markings on the screening mammograms, 90% (9/10 for cases with correct AI location and 18/20 for all) of invasive interval cancers were histologic grade 2 or 3.

Exploring the reviewed cases by time from screening to diagnosis, 90% (17/19) of true negative cases were diagnosed 13–24 months after a negative screening examination (Supplemental Table 4).

## Discussion

In this informed, consensus-based radiological review of 46 DBT interval breast cancers, radiologists classified 17% as false negative, 22% as minimal-sign significant, 20% as minimal-sign non-specific, and 41% as true negative. Overall, 35% of the cases with intermediate or high-risk AI scores (score 8–10) were marked with correct location by AI. However, only cases classified as false negative and minimal-sign significant were considered potentially detectable at AI-assisted screening rather than true-negative interval cancers, due to mammographic visibility.^20,22^ Considering only false negative cases, 11% of interval cancers were correctly marked by AI, whereas including both false negative and minimal-sign significant cases increased this proportion to 22%.

A systematic review of radiological reviews of interval cancers on DM screening reported that the proportion of false negative cancers ranged from 4 to 40% depending on review method.^15^ A prior retrospective DBT review reported 5% false negative and 11% minimal-sign significant cases (1/19 and 2/19, respectively), although the sample was limited to 19 interval cancers.^16^ Our finding of 17% false negative cases aligns with the results from previous DM-based retrospective reviews.^9,10,15^

In a study including data from a screening setting, using an earlier version of the AI model for DM and considering only cases with an AI score of 10, 78% of interval cancers were correctly localized in CC and/or MLO.^41^ In our study, this proportion was 65%, though only 17 cases had an AI score of 10, compared to 120 cases in the other study.^41^ Among cases with an AI score of 10, 24% were classified as false negative in our study, versus 11% in the other study.^41^ Furthermore, only one case (2%) classified as true negative by radiologists was correctly marked by AI in our study, whereas the earlier version of the AI model correctly marked 31% (37/120) of true negatives in DM screening.^33^ This aspect coupled with the less favorable histopathologic tumor characteristics of the true negative cases and diagnosis at 13–24 months after screening suggests that most of the true-negative interval cancers in our cohort may represent fast-growing tumors that were not visible at DBT screening.

A recent study by Yu et al., using an earlier version of the same AI model for DM and DBT, demonstrated that AI most frequently assigned intermediate or high scores (score 8–10) to missed interval cancers due to a reader error (68%, 13/19) and minimal-sign actionable cases (62%, 18/29).^20^ A study by Lång et al., also using an earlier version of the same AI model for DM, reported that 58% (83/143) of false negative or minimal-sign cases were accurately localized by AI, corresponding to a potential 19% reduction in interval breast cancers.^22^ A recent radiological review by Bahl et al. using a different AI model for DBT found that 33% (73/224) of interval cancers were correctly localized by AI retrospectively.

Yu et al. also showed that AI more accurately localized mammographically visible lesions compared to invisible ones.^20^ This is corroborated in our study, where the majority of false negative cases correctly marked by AI were classified as masses (7/8). This finding emphasizes the importance of lesion-level accuracy as a metric for evaluation of AI performance in DBT screening.^17,20^ Our results support the potential of AI to enhance DBT screening sensitivity by assisting radiologists in decision-making. This is consistent with prospective DM studies demonstrating that AI facilitates the detection of additional clinically significant cancers that might otherwise be overlooked by radiologists.^19,42^

In our sample, AI markings for cases with AI scores of 8–10 were incorrect in 24% of all cases (11/46), four of those cases were among false negative and minimal-sign significant, and six among minimal-sign non-specific and true negative. The cases with incorrect AI markings can be considered AI false positive cases and should be reduced, as these might result in unnecessary recalls. However, it is possible that due to those recalls the cancers in the true cancer locations could be diagnosed, resulting in the positive clinical value of the incorrect AI markings for examinations with high AI scores.^43^

The radiological review of screening mammograms showed that most of the masses in the false negative group had “equal” density. However, “high”-density mass patterns are more common for cancer cases.^33^ “Equal”-density masses could be difficult to detect resulting in reader errors. Interestingly, at the review of diagnostic mammograms, an opposite distribution for mass density was observed with the majority of masses having “high” density implying that density of the masses might increase over time. The comparisons between “high”- and “equal”-density masses and between screening and diagnostic mammograms should be interpreted with caution due to limited number of cases. An earlier detection of “equal”-density masses could be achieved by using contrast enhanced mammography or MRI.^44,45^

### Strengths and limitations

The study leveraged a population-based breast cancer screening program with high completeness of histologically verified cases.^34^ Radiologists performed the review blinded to AI markings and scores, preventing influence on their assessments. However, the main limitation is the small sample size. The proportions and possible trends should thus be interpreted with caution. The review was conducted by three internal breast radiologists involved in To-Be 2, which might represent introduce potential bias. However, representatives from the Cancer Registry, Norwegian Institute of Public Health were present during the review and responsible for data recording, minimizing the risk of interpretation bias. Mammographic features corresponding to AI markings were not documented, thereby limiting our ability to compare radiologists’ assessments with the AI model. The AI “match” criterion in this study might be considered overly restrictive, and a graded matching system including laterality, quadrant, and exact match, could provide a more nuanced assessment of AI’s potential clinical utility. Furthermore, potential false positives associated with the AI model performance in a screening setting were not investigated, as the study was focused on cancer detection. The use of a single mammography vendor and a single AI model limited generalizability of the findings. Comparisons across different versions of the same AI model should be interpreted with caution, as model performance may vary substantially between versions.^46^

In conclusion, 17% of interval cancers following DBT screening were classified as false negative in this retrospective consensus-based review. Using AI as decision support, 11–22% of interval cancers could potentially be detected earlier, highlighting the value of AI in improving DBT screening sensitivity.

## Supplemental material

Supplemental material - Missed and true interval cancers on digital breast tomosynthesis screening mammograms: radiologists’ assessment and artificial intelligence markings Supplemental material for Missed and true interval cancers on digital breast tomosynthesis screening mammograms: radiologists’ assessment and artificial intelligence markings by Nataliia Moshina, Marthe Larsen, Hildegunn S Aase, Kerstin Obst-Gleditsch, Ingrid Margrethe Ohm Ulvik, Hauke Bartsch, Leif Oltedal, and Solveig Hofvind in Acta Radiologica Open

## Data Availability

Data sharing requires legal basis and conditions set out in the General Data Protection Regulation (GDPR) and applicable national law and regulations. The dataset used for this study is not publicly available due to the conditions set out in GDPR and Norwegian law. Data from the Norwegian mandatory national health registries can be accessed via application to Helsedata. no.*
