# Supplementary material for: Missed and true interval cancers on digital breast tomosynthesis screening mammograms: radiologists’ assessment and artificial intelligence markings
Source: Acta Radiol Open. 2026 Aug 7;15(8):20584601261467016. doi: 10.1177/20584601261467016 (PMC13451661; doi:10.1177/20584601261467016)
Supplement: Supplementary file 1 [file sj-pdf-1-arr-10.1177_20584601261467016.pdf]

Supplementary Table 1. Mammographic features identified during the radiological review of screening and diagnostic mammograms for interval cancers classified as true negative, minimal-sign non-specific, minimal-sign significant, and false negative

Reviews	True negative n (%)	Minimal-sign non-specific n (%)	Minimal-sign significant n (%)	False negative n (%)
Screening mammograms	n=19	n=9	n=10	n=8
Calcification	-	-	2 (20%)	0
Architectural distortion	-	-	2 (20%)	0
Asymmetry	-	-	3 (30%)	1 (13%)
Mass	-	-	3 (30%)	7 (87%)
Negative	19 (100%)	9 (100%)	-	-
Diagnostic mammograms	n=19	n=9	n=10	n=8
Calcification	3 (16%)	0	1 (10%)	0
Architectural distortion	3 (16%)	3 (33%)	1 (10%)	0
Asymmetry	1 (5%)	1 (11%)	2 (20%)	0
Mass	10 (53%)	5 (56%)	5 (50%)	8 (100%)
Negative	2* (10%)	-	1* (10%)	-

* Two occult cases were in the true negative group and one case that did not have available images at diagnosis was in the minimal-sign significant group

Supplementary Table 2. Shape, margins and density of the cases classified as "masses" during the radiologic review of screening and diagnostic mammograms for true negative, minimal-sign non-specific, minimal-sign significant, and false-negative interval cancers.

Review	True negative* n (%)	Minimal-sign non-specific n (%)	Minimal-sign significant n (%)	False negative n (%)
Screening mammograms	-	-	n=3	n=7
Shape				
Irregular			1 (33%)	5 (71%)
Oval			1 (33%)	2 (29%)
Round			1 (33%)	0
Margins				
Circumscribed			1 (33%)	1 (14%)
Indistinct			0	1 (14%)
Obscured			1 (33%)	0
Microlobulated				0
Spiculated			1** (33%)	5 (71%)
Density				
High			2 (67%)	2 (29%)
Equal			1 (33%)	5 (71%)
Low			0	0
Fat-containing			0	0
Diagnostic mammograms	n=10	n=5	n=5	n=8
Shape				
Irregular	3 (30%)	4 (80%)	3 (60%)	7 (88%)
Oval	6 (60%)	1 (20%)	1	0
Round	1 (10%)	0	1	1 (12%)
Margins				
Circumscribed	3 (30%)	0	0	0
Indistinct	3 (30%)	1 (20%)	2 (40%)	0
Obscured	0	1 (20%)	0	0
Microlobulated	1 (10%)	0	1 (20%)	0
Spiculated	3 (30%)	3 (60%)	2 (40%)	8 (100%)
Density				
High	8 (80%)	2 (40%)	5 (100%)	6 (75%)
Equal	1 (10%)	3 (60%)	0	2 (25%)
Low	1 (10%)	0	0	0
Fat-containing	0	0	0	0

* Two occult cases were in the true negative group and one case that did not have available images at diagnosis was in the minimal-sign significant group

** This spiculated mass was classified as minimal-sign significant and not false negative by radiologists as it was very small and could be mistaken for crossing vascular structures

Supplementary Table 3. Histopathological tumor characteristics of A) invasive interval cancers classified as true negative, minimal-sign non-specific, minimal-sign significant, and false negative during the radiological consensus review of screening and diagnostic mammograms (n=46) and B) invasive interval cancers with available artificial intelligence (AI) markings for screening examinations prior to interval cancer (n=27)

	A) Interval cancers at the radiological review					B) Interval cancers with available AI markings		
	True negative N=19	Minimal-sign non-specific N=9	Minimal-sign significant N=10	False negative N=8	Total N=46	Correct location by AI, N=16	Incorrect location by AI, N=11	Total N=27
Ductal carcinoma in situ, n (%)	2 (11%)	1 (11%)	0	1 (13%)	4 (9%)	3 (19%)	0	3 (11%)
Invasive cancers, n (%)	17 (89%)	8 (89%)	10 (100%)	7 (87%)	42 (91%)	13 (81%)	11 (100%)	24 (89%)
Histopathological characteristics of invasive cancers								
Tumor diameter, median (IQR) mm	15 (8-35)	15 (2-25)	22 (15-36)	20 (9-30)	17 (9-30)	25 (15-39)	22 (15-25)	23 (15-38)
Data not available*, n	2	1	2	0	5	1	2	3
Histologic grade, n (%)								
Grade 1	0	2 (25%)	0	1 (20%)	3 (8%)	1 (10%)	1 (10%)	2 (10%)
Grade 2	11 (72%)	5 (62%)	5 (62%)	4 (80%)	25 (69%)	7 (70%)	8 (80%)	15 (75%)
Grade 3	4 (28%)	1 (13%)	3 (38%)	0	8 (22%)	2 (20%)	1 (10%)	3 (15%)
Data not available*, n	2	-	2	2	6	3	1	4
Lymph node involvement, n (%)	3 (19%)	0	4 (44%)	1 (14%)	8 (20%)	3 (25%)	4 (40%)	7 (32%)
Data not available*, n	1	-	1	-	2	1	1	2
Estrogen receptor positive (>1%), n (%)	14 (82%)	7 (88%)	8 (80%)	7 (100%)	36 (86%)	13 (100%)	10 (91%)	23 (96%)
Data not available*, n	-	-	-	-	-	-	-	-
Progesterone receptor positive (≥10%), n (%)	11 (65%)	5 (62%)	5 (50%)	7 (100%)	28 (67%)	9 (69%)	9 (82%)	18 (75%)
Data not available*, n	-	-	-	-	-	-	-	-
Immunohistochemical subtypes, n (%)								
Luminal A	7 (44%)	7 (88%)	5 (50%)	4 (67%)	23 (58%)	9 (75%)	7 (70%)	16 (73%)
Luminal B HER2 negative	3 (19%)	0	1 (10%)	0	4 (10%)	1 (8%)	2 (20%)	2 (14%)
Luminal B HER2 positive	2 (12%)	0	1 (10%)	2 (33%)	5 (12%)	2 (17%)	0	2 (9%)
HER2 positive	0	0	1 (10%)	0	1 (2%)	0	0	0
Triple negative	4 (25%)	1 (12%)	2 (20%)	0	7 (18%)	0	1 (10%)	1 (5%)
Data not available*, n	1	-	-	1	2	1	1	2

IQR – interquartile range

HER2 – human epidermal growth factor receptor 2

* Data not available due to neoadjuvant treatment or other reasons

Supplementary Table 4. Interval cancers classified as true negative, minimal-sign non-specific, minimal-sign significant, and false negative during the radiological review of screening and diagnostic mammograms stratified by months after breast cancer screening (≤12 months versus 13-24 months) A) For all interval cancers assessed in the review (n=46); B) For interval cancers with correct location of the artificial intelligence (AI) marking on mammograms (n=16)¤

Time between screening and diagnosis (months)	True negative	Minimal-sign non-specific	Minimal-sign significant	False negative	Total
A)					
≤12, n (%)*	2 (10%)	0	4 (40%)	5 (62%)	11 (24%)
13-24, n (%)*	17 (90%)	9 (100%)	6 (60%)	3 (38%)	35 (76%)
Total	19 (100%)	9 (100%)	10 (100%)	8 (100%)	46 (100%)
B)					
≤12, n (%)*	0	0	2 (40%)	2 (40%)	4 (25%)
13-24, n (%)*	1 (100%)	5 (100%)	3 (60%)	3 (60%)	12 (75%)
Total	1 (100%)	5 (100%)	5 (100%)	5 (100%)	16 (100%)

¤ Correct location of the AI marking in craniocaudal and/or mediolateral oblique views for mammograms with AI risk scores of 8-10

*Total numbers for each review classification category were used as denominators
